# EMERGENCY SURGERY FOR OBSTRUCTING COLON CANCER: MORBIDITY AND RISK FACTORS OF EARLY POSTOPERATIVE MORTALITY – A COHORT STUDY OF 118 CASES

**DOI:** 10.1590/0102-672020220002e1706

**Published:** 2023-01-09

**Authors:** Haithem ZAAFOURI, Mona CHERIF, Nizar KHEDHIRI, Meryam MESBAHI, Helmo ZEBDA, Raja JOUINI, Hedia BELLALI, Anis BEN MAAMER

**Affiliations:** 1 University Tunis El Manar, Faculty of Medicine of Tunis, Habib Thameur Hospital, General Surgery – Tunis;; 2 University Tunis El Manar, Faculty of Medicine of Tunis, Habib Thameur Hospital, Cytopathology – Tunis;; 3 University Tunis El Manar, Faculty of Medicine of Tunis, Habib Thameur Hospital, Epidemiology and Public Health – Tunis.

**Keywords:** Emergency Treatment, Mortality, Indicators of Morbidity and Mortality, Colonic Neoplasms, Tratamento de Emergência, Mortalidade, Indicadores de Morbimortalidade, Neoplasias do Colo

## Abstract

**BACKGROUND:**

Occlusion is the most common complication of colon cancer. Surgical treatment is associated with the highest morbidity and mortality rate (10–27%) and has the worst prognosis. It is necessary for immediate management, avoiding colic perforation and peritonitis. The increase in mortality in emergency colon cancer surgery is multifactorial.

**AIMS:**

The aim of this study was to identify the risk factors for early postoperative mortality that highlights the therapeutic strategy in the management of obstructive colon cancer.

**METHODS:**

A retrospective study was performed on patients admitted from 2008 to 2020 at the Department of General Surgery due to obstructive colon cancer and operated on as an emergency (within 24 h of admission).

**RESULTS:**

In all, 118 patients with colon cancer were operated, and the early postoperative mortality was 10.2%. The univariate analysis highlighted that the American Society of Anesthesiology score III or IV, perforation tumor, one postoperative complication, and two simultaneous postoperative complications were considered significant risk factors for early postoperative mortality after emergent surgery. Multivariate analysis showed that only tumor perforation and the occurrence of two postoperative complications were significant risk factors.

**CONCLUSION:**

This study showed that postoperative complication is the leading cause of early postoperative mortality after emergency surgery for obstructive colon cancer. Optimizing the postoperative management of these higher risk patients is still necessary and may reduce the mortality rate.

## RESUMO

**RACIONAL:**

A oclusão é a complicação mais comum do câncer de cólon. A cirurgia está associada à elevada morbimortalidade (10–27%) e pior prognóstico. É necessário indicação imediata, evitando perfuração cólica e peritonite. O aumento da mortalidade na cirurgia de emergência do câncer de cólon é multifatorial.

**OBJETIVOS:**

Identificar os fatores de risco de mortalidade pós-operatória precoce que levaram a destacar a estratégia terapêutica no manejo do câncer de cólon obstrutivo.

**MÉTODOS:**

Estudo retrospectivo em pacientes admitidos no Departamento de Cirurgia Geral, entre 2008 e 2020, por câncer de cólon obstrutivo e operados de emergência (dentro de 24 horas da admissão).

**RESULTADOS:**

Foram operados 118 pacientes e a mortalidade pós-operatória precoce foi de 10,2%. A análise univariada destacou que escore American Society of Anesthesiology III ou IV, tumor perfurado, uma complicação pós-operatória e duas complicações pós-operatórias simultâneas foram considerados fatores de risco significativos de mortalidade pós-operatória precoce após cirurgia de emergência no câncer de cólon obstrutivo. Na análise multivariada, apenas a perfuração tumoral e a ocorrência de duas complicações médicas pós-operatórias foram fatores de risco significativos.

**CONCLUSÃO:**

Este estudo mostrou que a complicação pós-operatória é a principal causa de mortalidade pós-operatória precoce após cirurgia de emergência de câncer de cólon obstrutivo. Otimizar o manejo pós-operatório desses pacientes de alto risco ainda é necessário e pode reduzir a taxa de mortalidade.

## INTRODUCTION

The majority of colorectal cancer patients present with surgical emergencies, the most common being obstruction and perforation. This surgery has the worst prognosis and is associated with the highest morbidity and mortality rate (10–27%)^6^.

The appropriate management of complicated colon cancer poses the following problems:

- Consequences of obstruction on the intestine
- The stage that is often locally advanced and/or metastatic
- Unfavorable medical history of patients

The objective of this study was to analyze the postoperative morbidity and mortality in patients with obstructed colon cancer who had emergency surgery and to identify the risk factors for early postoperative mortality that highlights the therapeutic strategy in the management of obstructive colon cancer.

## METHODS

A retrospective observational and analytic study was conducted on patients who had emergency surgery (within 24 h of admission) for obstructing colon cancer at the Department of Surgery, Habib Thameur Hospital, from January 2008 to December 2020. The Ethics Committee of the Habib Thameur Hospital approved the study.

Data were obtained from a retrospectively maintained institutional database including age, gender, tumor location (more than 15 cm from the anal margin), type of surgery, hospital stay postoperative morbidity, and mortality. Postoperative mortality was defined as the occurrence of death within 30 days of surgery.

The Fisher’s exact test was used to determine the differences between the variables and calculate univariate continuous variables in order to predict factors of early postoperative mortality. The Cox proportional model was used for survival analysis and multivariate study. A p-value <0.05 was considered statistically significant.

## RESULTS

From January 2008 to December 2020, 118 patients were operated on for colon cancer (66 males and 52 females; male-to-female ratio of 1:2). The average age was 66 years (34–92 years). Overall, 50 (42.4%) patients were older than 70 years of age. Almost half (47.4%) of our patients had a body mass index (BMI) of 20 kg/m^2^, and 38.1% of patients had anemia with hemoglobin (HB) ≤10 g/dl. All patients were evaluated by an anesthetic physician and then classified according to the classification American Society of Anesthesiology score (ASA): 86.4% were ASA I or II, and 13.6% were ASA III or IV.

The tumor was mainly located in the sigmoid colon (65.2% of all cases), and a perforated tumor was found in 6.8% of cases (Table 1).

**Table 1:** Characteristics of patients

Characteristics of patients	Number of patients	%
Gender		
M	66	55.9
F	52	44.1
Age (years)		
≥70	50	42.4
<70	68	57.6
BMI ≤20	56	47.4
Hb ≤10	45	38.1
ASA grade		
ASA I+II	102	86.4
ASA III+IV	16	
Tumor site		
Sigmoid colon	77	65.2
Descending colon	**17**	
Right colon	21	17.8
Transverse colon	3	2.6
Tumor perforation	8	6.8
Surgery		
Palliative	17	14.4
Curative	101	85.6
Total or subtotal colectomy	34	28.8
Anastomosis after resection	67	56.8
Intraoperative blood transfusion	12	10.1

ASA: American Society of Anesthesiology score.

All patients were operated on by a senior surgeon, with a median laparotomy in all cases.

The type of surgery was performed concerning the patients’ characteristics and comorbidities, the clinical presentation, and the preoperative findings. Curative surgery was performed in 85.6% of cases.

A total or subtotal colectomy was carried out in 28.8% of cases. Primary anastomosis after colon resection was achieved in 56.8% of cases. Ten percent of patients required a preoperative blood transfusion. Anastomotic dehiscence occurred in 8 (11.9%) out of 67 patients with primary anastomosis.

Postoperative medical complications such as sepsis, pulmonary embolism, and heart or kidney failure were noted in 31.4% of cases, and 11% had two complications at the same time. The early postoperative mortality was 10.2%.

We looked at age over 70 years, male sex, BMI ≤20 kg/m^2^, HB ≤10 g/dl, ASA score III or IV, tumor perforation, palliative surgery, sub or total colectomy, preoperative blood transfusion, anastomotic leakage, a postoperative complication, and two simultaneous postoperative complications as risk factors for early postoperative mortality.

The univariate analysis highlighted that ASA III or IV score, perforation tumor, a postoperative complication, and two simultaneous postoperative complications were considered significant risk factors of early postoperative mortality after emergency surgery of obstructive colon cancer (Table 2).

**Table 2:** Univariate analysis of factors associated with death within 30 days after surgery.

Characteristics of patients	Number of patients	Number of death within (%)	Odds ratio	p-value (%)
Total	118 (100)	12 (10.2)		
Age ≥70 years 0.121	50 (42.4)	8 (16)	–	
Gender M 1.00	66 (55.9)	7 (10.6)	–	
BMI ≤20	56 (47.4)	–	–	
Hb ≤10	45 (38.1)	–	–	
ASA III+IV	16 (13.6)	4 (25)	**3.9 [1.0–14.9]**	**0.035**
Tumor perforation	8 (6.8)	4 (50)	**12.7[2.6–60.7]**	**0.000**
Palliative surgery	17 (14.4)	2 (11.8)	–	0.814
Total or subtotal colectomy	34 (28.8)	4 (11.8)	–	0.715
Intraoperative blood transfusion	12 (10.1)	–	–	
Anastomosis after resection	67 (56.8)	6 (9)	–	0.761
Anastomotic leak	8 (11.9)	2 (25)	–	0.09
Postoperative medical complications	37 (31.4)	11 (29.7)	**33.8 [4.1–274.8]**	**0.000**
Postoperative two medical complications	13 (11)	8 (61.5)	**40.4 [9.0–180.8]**	**0.000**

ASA: American Society of Anesthesiology score.

Finally, postoperative complications were the main cause of postoperative mortality: the mortality was 29.7% in “the one postoperative complication group” with p<0.05 and odds ratio (OR) of 33.846 and was 61.5% in ‘’the two postoperative complications group’’ with p<0.05 and OR of 40.4. In the multivariate analysis, only tumor perforation and the occurrence of two postoperative complications were significant risk factors for early postoperative mortality after emergency surgery of obstructing colon cancer.

## DISCUSSION

Occlusion is the most common complication of colon cancer. Perforation causing severe sepsis (due to peritonitis and bacterial translocation) can occur due to this acute obstruction, which accounts for prompt management with a double challenge: on the one hand, the challenge is to remove the occlusion quickly, avoiding colic perforation and peritonitis; on the other hand, the outcome of surgery is to provide curative management with oncology standards, under appropriate circumstances, according to the locoregional and general conditions of the patient^9^.

While several studies have analyzed the prognosis of occlusive colon cancer, studying the morbimortality and the long-term oncological outcomes, very few studies have investigated the risk factors of early postoperative mortality after emergency surgery for this etiology of intestinal obstruction.

Emergency surgery is more associated with increased postoperative morbidity, which is estimated between 38 and 50% versus 20 and 30% in elective surgery^2,8^. Mortality is higher in emergencies, ranging from 10 to 20%, than in elective surgery (3–6%)^2,9^, regardless of the type of surgery performed: curative or palliative^8^.

The increase in mortality in emergency surgery for colon cancer is multifactorial. First, it is associated with the alteration of general conditions of patients, whose rate of ASA scores III and IV ranges from 40 to 50%^4,9^. Two recent studies (one in Denmark and the second published by the French Surgical Association), involving more than 2000 patients, demonstrated that 35% of patients were classified as ASA scores III and IV^5,7^. Patients operated in an emergency are older than patients operated electively: 49% were older than 75 years in the emergency group versus 39% in the elective group^3^. The high ASA score is considered an independent risk factor for postoperative mortality in emergent general surgery or surgery of occlusive colon cancer, in which an ASA score II multiplies the risk of postoperative death by 3.22, an ASA score III multiplies the risk of postoperative death by 11.73, and an ASA score IV multiplies by 22.33 compared to ASA score I^9^. In our series, the high ASA score was a significant risk factor for early postoperative mortality: ASA scores III and IV were associated with 25% of mortality compared to 7.8% in ASA scores I and II with an OR of 3.9, but this result was not found in the multivariate study due to the low effectiveness.

Patients aged between 65 and 70 years had a higher mortality rate with a risk of postoperative death multiplied by 2.97 than in patients under 65 years. Furthermore, patients older than 75 years have a risk of death multiplied by 4.31^9^.

This risk factor was studied by the French Association of Surgery in 2019^7^. In a 2018 Swedish study^1^ treating more than 3000 patients undergoing emergency surgery for complicated colic cancer, instead of 94% being operated on for occlusion or perforation, concluded that the early postoperative mortality was 10.5%. In multivariate analysis, this study preserved elderly age, ASA score, and perforated tumor as risk factors of postoperative mortality.

Four independent risk factors of postoperative mortality after emergency surgery for occlusive colon cancer were identified by the French Association of Surgery in 2002^2^: age more than 70 years, emergent colectomy, neurological history, and recent weight loss.

The main risk factor of postoperative mortality was the occurrence of medical complications such as cardiopulmonary, renal, thromboembolic, and infectious complications; 24.4% of patients developed one medical complication and had a mortality rate of 57.8% at 30 days^5^. The mortality of patients without postoperative complications increased from 8.5 to 39.4% in patients with one complication and 47.4% in patients with two complications. The specific surgical complications (20.4%) had no significant impact on mortality. In the multivariate study, the other risk factors for postoperative mortality were age more than 70 years, male sex, ASA score more than III, palliative intervention, a perforated tumor, splenectomy, and preoperative complication.

## CONCLUSIONS

This study showed that medical postoperative complication is the main cause of early postoperative mortality after emergency surgery for obstructive colon cancer. Optimized postoperative management of these higher risk patients is still required and can reduce the mortality rate.
